# Relating Cultural Distance to Self-Other Agreement of Leader–Observer Dyads: The Role of Hierarchical Position

**DOI:** 10.3389/fpsyg.2021.738120

**Published:** 2021-10-21

**Authors:** Tim Vriend, Caroline Rook, Harry Garretsen, Janka I. Stoker, Manfred Kets de Vries

**Affiliations:** ^1^International Business School, Hanze University of Applied Sciences, Groningen, Netherlands; ^2^Faculty of Economics and Business, University of Groningen, Groningen, Netherlands; ^3^Henley Business School, Henley-on-Thames, United Kingdom; ^4^INSEAD, Paris, France

**Keywords:** cultural distance, self-other agreement, leadership categorization theory, approach/inhibition theory of power, multisource feedback systems

## Abstract

Multisource feedback is important for leadership development and effectiveness. An important asset of such feedback is that it provides information about the self-other agreement between leaders and observers. Self-other agreement relates to several positive individual, dyadic, and organizational outcomes. Given the increasingly intercultural context in organizations, it is imperative to understand whether and how cultural distance between leaders and observers relates to self-other agreement. We hypothesize that cultural distance within leader-observer dyads is negatively associated with self-other agreement. Moreover, we expect that this relationship is stronger for leader-superior than leader-subordinate dyads. We use a unique multi-cultural dataset of 7,778 leaders (52 nationalities) rated by 22,997 subordinates (56 nationalities) and 10,132 superiors (54 nationalities) to test our hypotheses. Results confirm that cultural distance is negatively associated with self-other agreement; we show that this relationship is driven by increased self-ratings and by reduced other-ratings. In addition, we find that these results are more pronounced for leader–superior than for leader–subordinate dyads. Implications for the theory and practice of self-other agreement and multisource feedback are discussed.

## Introduction

In the contemporary, globalized and interconnected business world, where the number of intercultural interactions between people are ever increasing, practicing effective leadership has never been more challenging (Maddux et al., 2021). Accordingly, leaders are increasingly relying on multisource feedback (or 360° feedback) from subordinates, peers, supervisors, and/or customers has become to improve their leadership effectiveness (Atwater et al., 1998, 2002). Indeed, research into multisource feedback suggests that self-other agreement is especially desirable for leaders: notwithstanding boundary conditions, leaders with high self-other agreement have superior individual, dyadic, and even organizational outcomes (Van der Kam et al., 2014).

Self-other agreement is the extent to which leaders’ perceptions of their own leadership behaviors align with the perceptions of observers, like subordinates and superiors (Atwater et al., 1998). Research shows that disagreement is generally more likely to occur than agreement (Harris and Schaubroeck, 1988; Tsui and Ohlott, 1988; Heidemeier and Moser, 2009; Lee and Carpenter, 2018), especially when leaders and observers hold different perceptions about the evaluation criteria used (Tsui and Ohlott, 1988; Yammarino and Atwater, 1993; Oh and Berry, 2009). These criteria are, at least partly, based on people’s cultural backgrounds, which has important implications for how people conceptualize effective leadership practices (Den Hartog et al., 1999; House et al., 2004) and how people evaluate and provide feedback (Atwater et al., 2005, 2009; Gentry et al., 2007; Varela and Premeaux, 2008; Eckert et al., 2010). When people are culturally similar – that is, their *cultural distance* is negligible (Shenkar, 2001) – they are likely to hold similar views on appropriate and effective leadership behaviors and thus evaluation criteria. As the cultural distance between people increases, however, the number of values, beliefs, and norms they share become decisively fewer, which leads them to have increasingly different views on appropriate and effective leadership behaviors (House et al., 2002; Dickson et al., 2003). Although we would argue that such different views would also feed into the (mis)alignment of leaders and their culturally distant observers, to the best of our knowledge, empirical research studying this is limited. There is research demonstrating that the extent and form of self-other agreement differs *between* countries and cultures (e.g., Atwater et al., 2005; Gentry et al., 2007; Varela and Premeaux, 2008), but this line of research has solely studied dyads from the *same* countries and/or cultures. Moreover, previous research has established that coming from different countries and cultures can indeed influence evaluations in general (e.g., Caligiuri and Day, 2000; Kossek et al., 2017), but never within the context of (dis)agreement in evaluations. Accordingly, more research on the link between cultural distance and self-other agreement is warranted.

In this study, we investigate the extent to which cultural distance relates to self-other agreement of transformational and transactional leadership behaviors (Burns, 1978; Bass, 1985; Bass and Avolio, 1990), which we respectively define as the extent to which leaders articulate a compelling vision, mission, and strategy (i.e., transformational), and the extent to which leaders set up appropriate reward structures and provide feedback (i.e., transactional) (cf. Kets De Vries et al., 2004). We present two hypotheses to argue why and when cultural distance relates to self-other agreement. First, drawing from leadership categorization theory (Lord et al., 1982), which holds that individuals attribute enhanced effectiveness to leaders that act in line with their own leader prototypes (van Knippenberg, 2011; Lord et al., 2020), we hypothesize that cultural distance is negatively related to self-other agreement. Second, imbuing this leadership categorization theory perspective with insights from the approach/inhibition theory of power (Keltner et al., 2003), which describes that those with high power (i.e., superiors) are more likely than those with low power (i.e., subordinates) to think and act in line with their own values (Keltner et al., 2003; Galinsky et al., 2008), we hypothesize that the negative relationship between cultural distance and self-other agreement will be stronger for leader-superior dyads than for leader-subordinate dyads. We test these two hypotheses using a unique, longitudinal database that covers the period from 2003 to 2012, containing managers, subordinates and superiors that evaluate manager’s behaviors (see Agrawal and Rook, 2014). This dataset includes 7,778 leaders (52 nationalities) rated by 22,997 subordinates (56 nationalities) and 10,132 superiors (54 nationalities) who completed a leadership inventory (the GELI; see Kets De Vries et al., 2004). Following previous research on self-other agreement (Ostroff et al., 2004; McKee et al., 2018), we used a multivariate regression approach to estimate the effects on self-other agreement (Edwards, 1995).

Our study has two important contributions. First, it answers calls for more research on the role of culture in multisource feedback for leadership (Varela and Premeaux, 2008; Fleenor et al., 2010; Kossek et al., 2017) and studies on culture and leadership in general (Atwater et al., 2021). To the best of our knowledge, our study is the first to provide empirical evidence for the important role of cultural distance in leader–observer self-other agreement. Indeed, research on cultural distance is mostly limited to the international management literature (Hutzschenreuter et al., 2016; Beugelsdijk et al., 2017; Srivastava et al., 2020) and research on cultural distance within the context of leadership and self-other agreement is limited. Previous research has demonstrated that self-other agreement differs between countries and cultures (e.g., Atwater et al., 2005; Gentry et al., 2007; Varela and Premeaux, 2008) and that being from different countries and cultures can affect evaluations in general (e.g., Caligiuri and Day, 2000; Kossek et al., 2017). Continuing these streams of research, by integrating literature on self-other agreement (Yammarino and Atwater, 1993; Atwater et al., 1998) with leadership categorization theory (Lord et al., 1982, 1984; Lord and Maher, 1991), we show that cultural distance within leader–observer dyads is indeed negatively associated with self-other agreement. Second, our study contributes to the discussion that different types of observers should be considered when studying multisource feedback (Heidemeier and Moser, 2009; Lee and Carpenter, 2018). Previous empirical research has suggested that the effects of cultural distance on self-other agreement across different leadership behaviors may be contingent upon the hierarchical position of the observer (Caligiuri and Day, 2000; Kossek et al., 2017). By combining leadership categorization theory (Lord et al., 1982, 1984; Lord and Maher, 1991) with the approach/inhibition theory of power (Keltner et al., 2003), we corroborate and extend these findings, and show that especially the position of superiors is crucial when it concerns the role of cultural distance in self-other agreement.

## Theory and Hypotheses

### Self-Other Agreement

Leader’ self-other agreement reflects the extent to which leaders’ perceptions of their own behaviors converge and diverge from the perceptions of observers, including subordinates, peers, and superiors (Yammarino and Atwater, 1993; Atwater et al., 1998). Leaders’ self-ratings are only modestly correlated with observers’ ratings (weighted and corrected correlations between 0.14 and 0.56, Lee and Carpenter, 2018), suggesting that disagreement between leaders and observers is more likely to occur than agreement. Initial studies considered disagreement to be an indication of over- or underestimation on part of the leader, assuming that observer ratings are more accurate than leader ratings (Yammarino and Atwater, 1993; Atwater et al., 1998). A more current perspective, however, argues that not only leader’ but also observer’ ratings may be inaccurate (Fleenor et al., 2010).

Indeed, both leaders and observers may be prone to all sorts of (cognitive) biases that influence their self- and other-ratings. Leaders are prone to self-enhancement biases (Harris and Schaubroeck, 1988; Tsui and Ohlott, 1988; Yammarino and Atwater, 1993), which cause them to rate themselves higher than average, by emphasizing positive information and neutralizing negative information (Taylor and Brown, 1988), consequently inflating their self-ratings (Bass and Yammarino, 1991). Observers are also prone to several cognitive biases that influence their ratings, including central tendency, halo, leniency, restriction of range, and severity biases (Saal et al., 1980; Harris and Schaubroeck, 1988; Van Velsor et al., 1993). Consequently, leaders and observers may arrive at different ratings of leadership behaviors because of differences in observations and/or in criteria used (Tsui and Ohlott, 1988; Yammarino and Atwater, 1993; Oh and Berry, 2009).

Several scholars studied antecedents that activate the (cognitive) biases which underlie self-other (dis)agreement. In a review study, Fleenor et al. (2010) summarized how a wide variety of rater, ratee, and rater-ratee dyadic characteristics influence self-other agreement, including biographical characteristics (e.g., age and gender), personality and individual characteristics (e.g., Big Five traits), and job relevant experiences (e.g., feedback giving and receiving). Although they (Fleenor et al., 2010) noted national culture as an important characteristic that directly relates to self-other agreement, as we review in more detail below, evidence for the specific role of cultural distance between rater and ratee is scarce.

### Culture and Cultural Distance

We define culture as “characteristic patterns of social behavior, social interaction, and conscious and unconscious influences on action that recur in or typify a society,” with societies being “large, differentiated groups with which members share an identity throughout life” (Peterson and Barreto, 2014, p. 1134). Scholars have developed various typologies to capture these characteristic patterns through different cultural value dimensions (Hofstede, 1980, 2001; Schwartz, 1999; House et al., 2004). These cultural dimensions represent societal values on an aggregate level, which does not necessarily imply that every individual within that society adheres to or shares these values, because individuals may have personal values that are not at all aligned with the society they were cultivated in Hofstede (2006), Peterson and Castro (2006), e.g., ecological fallacy, Robinson (1950). However, according to the *cultural expertise and personal values* proposition as developed by Peterson and Barreto (2014), it can be argued that being cultivated in a specific societal culture does have a strong influence on both individual members’ cognitive structures (e.g., expertise and intuitive understanding) and acceptance of cultural values, regardless of whether they personally endorse these specific cultural values. Accordingly, for the purpose of our study, when we discuss culture or cultural values, we assume that societal cultural values have influenced how individuals from that culture think and act, although we acknowledge that their specific personal values may not be perfectly aligned.

In support of the cultural expertise and personal values proposition, research has established that leader- and observer-ratings are conditional upon cultural value dimensions (Atwater et al., 2005; Gentry et al., 2007; Fleenor et al., 2010), including assertiveness, individualism, humane orientation, and power distance (Varela and Premeaux, 2008; Atwater et al., 2009; Eckert et al., 2010; Kossek et al., 2017). Results of these studies show for instance that societal cultural values influence how leaders rate themselves (cf. the cultural-relativity hypothesis, Farh et al., 2007) and agreement between self- and other-ratings (Atwater et al., 2009; Eckert et al., 2010). However, research on the role of cultural *distance* between rater and ratee is limited.

As mentioned earlier, we define ‘cultural distance’ as the extent to which leaders and observers differ in their cultural values (Shenkar, 2001). Although any of the afforementioned typologies for culture can be used as input to define the extent of cultural distance, we have opted to focus on the nine GLOBE dimensions (assertiveness, gender egalitarianism, future orientation, humane orientation, in-group collectivism, institutional collectivism, performance orientation, power distance, and uncertainty avoidance) as they resonate best with our leadership context (House et al., 2004). Cultural distance is then the extent to which leaders and observers increasingly differ on such dimensions. The idea being that as cultural distance increases, leaders and observers are likely to hold increasingly different values, beliefs, and norms (Shenkar, 2001; Beugelsdijk et al., 2018).

Despite there being myriad studies on how culture *in general* relates to self-other agreement (see e.g., Atwater et al., 2009; Eckert et al., 2010), we know of no research studying the role of cultural distance (cf. Fleenor et al., 2010). There are a few studies that demonstrate the role of cultural distance (that is, being from a different nationality) for observer ratings. Caligiuri and Day (2000) demonstrated that superiors provide lower ratings of contextual and assignment-specific performance to subordinates from different nationalities. In a study of expatriates, Kossek et al. (2017) demonstrated that cultural distance is negatively related to subordinate- and peer-ratings of task and contextual leadership behaviors, but unrelated to superior-ratings. Furthermore, although not the focus of their study, Smith et al. (2012) demonstrate that transformational leadership suppresses the negative effect of cultural distance on relationship quality. Finally, Testa (2002, 2009) demonstrated that subordinates from different nationalities rate their leaders lower on consideration behaviors than culturally similar subordinates.

Although these studies certainly suggest that being from a different culture may influence observer ratings, they are not telling us anything about the possible relationship between cultural *distance* between the leader and the observer, and *self-other agreement*. In order to hypothesize how cultural distance could relate to self-other agreement, we draw upon leadership categorization theory.

### Leadership Categorization Theory

Leadership categorization theory (Lord et al., 1982, 1984; Lord and Maher, 1991) argues that people continually assess whether a certain person fits a leader role (for reviews, see Epitropaki et al., 2013; Offermann and Coats, 2018; Lord et al., 2020). According to leadership categorization theory, individuals construe implicit leadership theories that are cognitive representations of effective or ‘good’ leader prototypes in terms of personality, qualities, and traits (Lord et al., 1982, 1984; Lord and Maher, 1991). Individuals use these implicit leadership theories to determine whether individuals fit into a leader category, consequently selectively retrieving and encoding information about individuals that is consistent with their effective leader prototypes (Phillips and Lord, 1982; Rush and Russell, 1988; Kenney et al., 1994; van Knippenberg, 2011). Individuals with characteristics that match these effective leader prototypes are more likely to be categorized as leaders than individuals without (Cronshaw and Lord, 1987). Consequently, categorized leaders are more likely than non-categorized leaders to be attributed positive characteristics, including enhanced collegiality, responsibility, and causality for positive events (Lord et al., 1984; Nye and Forsyth, 1991; Ensari and Murphy, 2003), even if actual behavior is similar between prototypical and non-prototypical leaders (Schyns, 2006).

Implicit leadership theories are important pillars of the seminal GLOBE-studies (House et al., 2002, 2004), which studied how societal and organizational cultures affect leadership and organizational practices. In these studies, scholars argued that societal cultural values influence the implicit leadership theories that individuals have about effective leadership prototypes (for a recent overview, see Dorfman et al., 2012) – that is, individuals develop cultural expertise about what constitutes effective leadership. Indeed, the studies demonstrate substantial cross-cultural variation in implicit leadership prototypes and effects (Den Hartog et al., 1999; Dickson et al., 2003; House et al., 2004). Individuals across cultures differ substantially in their interpretations of and preferences for specific leadership behaviors (Gerstner and Day, 1994; Offermann and Hellmann, 1997; Lord and Emrich, 2000; Lord et al., 2001; Ensari and Murphy, 2003; Tsui et al., 2007). Following leadership categorization theory, leadership behaviors that are congruent with one’s societal cultural values are generally perceived to be more effective (Newman and Nollen, 1996) and leaders that demonstrate such culturally congruent behaviors are more likely to be perceived as prototypical (Shaw, 1990). A fit between culturally endorsed leadership prototypes and actual leader attributes and behaviors contribute to leader acceptance and effectiveness (House et al., 2014). Accordingly, leadership categorization theory and its implied implicit leadership theories are often used to explain effects on and of leadership in intercultural contexts (e.g., Dorfman et al., 2012).

One of the main purposes of the GLOBE studies was to explore which leadership attributes and behaviors were universally desirable (i.e., part of all cultures’ leadership prototypes) versus culturally contingent (i.e., part of some cultures’ leadership prototypes). Although the empirical evidence is relatively clear that certain leadership values are considered to be universally desirable, such as being trustworthy, just, and honest (Den Hartog et al., 1999; Dorfman et al., 2012), empirical evidence on the universality of leadership *behaviors* is mixed (Crede et al., 2019). Whereas some scholars claim that transformational (i.e., charismatic) and transactional (i.e., contingent reward) leadership may be universal (e.g., Dorfman et al., 1997), others claim that their assessment and manifestations may be culturally contingent (e.g., Money and Graham, 1999; Javidan and Carl, 2004; Karakitapoglu-Aygün and Gumusluoglu, 2013). Given that the GLOBE studies demonstrate significant influences of cultural values across all culturally endorsed leadership attributes (House et al., 2004; Dorfman et al., 2012), it seems feasible to assume that leadership behaviors are at least to some extent part of cultural implicit theories of effective leadership.

### Relating Cultural Distance to Self-Other Agreement

Based on self-other agreement literature, leadership categorization theory, and the GLOBE studies, we hypothesize that cultural distance is negatively related to self-other agreement. First, we argue that cultural distance by definition implies that leaders and observers have different prototypes of effective leadership. As mentioned earlier, culture reflects “characteristic patterns of social behavior, social interaction, and conscious and unconscious influences on action that recur in or typify a society” (Peterson and Barreto, 2014, p. 1134), captured along several cultural dimensions (Hofstede, 1980; Schwartz, 1999; House et al., 2004). Cultural distance reflects the extent to which cultures diverge or converge on these cultural dimensions. Moreover, as cultures become more distal, the number of values, beliefs, and norms shared between two cultures become decisively fewer (Shenkar, 2001). Furthermore, societies differ substantially in their leadership prototypes and thus what is expected of leaders in order for them to be effective (Offermann and Hellmann, 1997; Lord and Emrich, 2000; Lord et al., 2001; Ensari and Murphy, 2003; Tsui et al., 2007). The contents and manifestations of leadership behaviors are also culturally dependent, such that they are likely to be influenced by the nuances unique to their cultures’ leadership prototypes (Javidan and Carl, 2004; Karakitapoglu-Aygün and Gumusluoglu, 2013). Overall, therefore, as cultural distance increases, the shared ideas about effective leadership prototypes between leaders and observers become less, which makes agreement less likely.

Second, we argue that such leadership prototypes especially shape observers’ ratings of leaders’ behaviors. Rating involves a cognitive process where raters constantly observe, store, encode, integrate, and evaluate the leadership behaviors performed (DeNisi et al., 1984). As mentioned earlier, people are prone to a variety of cognitive biases during this rating process (Saal et al., 1980; Wherry and Barlett, 1982; Harris and Schaubroeck, 1988; Taylor and Brown, 1988; Van Velsor et al., 1993; Yammarino and Atwater, 1993). Importantly, people continuously rely on their leadership prototypes when evaluating leaders (Lord and Maher, 1991), such that raters attribute higher performance evaluations to leaders that act in line with these prototypes (Lord et al., 1984; Nye and Forsyth, 1991; Ensari and Murphy, 2003).

Third, combining the preceding two lines of argumentation, we argue that cultural distance will be negatively associated with self-other agreement, especially due to lower other-ratings. Cultural distance is likely to reduce the extent to which observers categorize leaders as such (Lord and Maher, 1991), which ultimately reduces their ratings of the observed leaders’ behaviors (Lord et al., 1984; Nye and Forsyth, 1991; Ensari and Murphy, 2003). Accordingly, we hypothesize that:

*Hypothesis 1: Cultural distance is negatively related to self-other agreement.*

### The Moderating Role of Hierarchical Position of the Observer

The role of cultural distance in self-other agreement may be contingent upon the hierarchical position of the observer, because several studies demonstrated that self-other agreement may differ substantially based on the hierarchical position of the observer relative to the leader (Lee and Carpenter, 2018). Different types of observers (that is, subordinates and superiors) often do not agree in their ratings of leaders’ behaviors (corrected correlations between 0.22 and 0.34, Conway and Huffcutt, 1997). This finding is typically explained by the fact that observers differ in the types of behaviors they observe, and the performance criteria they use (Tsui and Ohlott, 1988; Yammarino and Atwater, 1993; Oh and Berry, 2009). Also, leaders are argued to differentiate in their behaviors toward different observers (Tett and Burnett, 2003). Leaders target leadership behaviors associated with daily operations at their subordinates, and exert more strategic and political leadership behaviors toward their superiors (Den Hartog et al., 1999). Hence, different observers observe different leadership behaviors from the same leader (Hiller et al., 2011; Hansbrough et al., 2015; Lee and Carpenter, 2018), which in the end also relates to the self-other agreement for leader-subordinate versus leader–superior dyads.

Besides the fact that because of their hierarchical position, different observers might encounter different behaviors of the same leader, we argue that there is another major reason for differences in self-other (dis)agreement differences, namely power. According to the approach/inhibition theory of power (Keltner et al., 2003), the extent of power an individual has relative to others influences their affective, behavioral, cognitive, and motivational states (Keltner et al., 2003). Individuals with high power are more likely than those with low power to act in accordance with their own interests and values and to discard those of others (Keltner et al., 2003; Galinsky et al., 2006, 2008; Magee and Galinsky, 2008). Relative to those with low power, individuals with high power more selectively process information (Guinote, 2007) and are more likely to underestimate threats (Inesi, 2010) and overestimate their own abilities and performance (Brutus et al., 1999). Furthermore, individuals with high power are less receptive to feedback (Sully de Luque and Sommer, 2000; Atwater et al., 2009) and hold those with low power to stricter standards than they would themselves (Lammers et al., 2010).

Clearly, one’s position in a hierarchy determines the extent of their power, such that superiors have high power in leader–superior dyads, whereas subordinates have low power in leader–subordinate dyads. Following both the leadership categorization theory and the approach/inhibition theory of power, we argue that superiors are more likely than subordinates to rely on their implicit leadership prototypes when rating leadership behaviors. Consequently, this proposition has implications for our Hypothesis 1. There, we hypothesized that that cultural distance negatively relates to self-other agreement due to differences in leadership prototypes. We expect that this relationship between cultural distance and self-other agreement is more pronounced for leader-superior dyads, because superiors are more likely to act upon their culturally laden leadership prototypes. We therefore hypothesize that:

*Hypothesis 2: The negative relationship between cultural distance and self-other agreement is stronger for leader-superior than leader-subordinate dyads.*

## Materials and Methods

### Sample and Procedure

Data were gathered from 14,523 middle and top management executives who attended leadership development programs at an international business school (France, Singapore, and the Middle East) between 2003 and 2012. Because of this international business context, and because of the fact that this school is also based in three different locations, the sample of respondents is unique when it comes to the number of nationalities. This uniqueness allows for broad generalizations across multiple cultures, leaders, nationalities, and organizations. These executives filled out a survey in which they would indicate self-ratings of their own leadership behaviors. Furthermore, these executives were asked to nominate observers who would provide other-ratings on the same leadership behaviors. Being part of the leadership developmental program, executives were specifically instructed to only nominate those observers that can provide honest feedback, lest they would receive invalid responses that would hamper their development. The observers did not provide personal identifying information and subordinate’s responses were completely anonymous. A total of 41,027 subordinates and 17,259 superiors provided such other-ratings. A link to the online survey was sent to the executive and their observers several weeks before the executive took part in the leadership development program. Both self- and other-ratings were provided before the executives attended the leadership development program. Part of this dataset has been used in previous publications (Ibarra and Obodaru, 2009; Agrawal and Rook, 2014).

We took a three-step approach to preparing the data for analysis. First, we had to account for the fact that several leaders and observers participated multiple times. Particularly, several observers (mostly superiors) have provided multiple ratings of (different) leaders. Providing multiple ratings may be associated with various cognitive biases, including central tendency, halo, leniency, restriction of range, and severity biases (Saal et al., 1980; Wherry and Barlett, 1982; Harris and Schaubroeck, 1988; Taylor and Brown, 1988; Van Velsor et al., 1993; Yammarino and Atwater, 1993). To ensure that ratings are unaffected by such cognitive biases, we limited ourselves to the chronogically first self- or other-rating that participants provided, reducing our sample to 14,209 leaders, 39,955 subordinates, and 13,743 superiors. Second, we removed participants with missing values on our focal variables, further reducing our sample to 12,190 leaders, 34,564 subordinates, and 11,996 superiors. Third, to enable us to assess differences between subordinates and superiors (cf. Hypothesis 2), we only retained leaders for which we had self-, subordinate-, and superior-ratings.

Our final sample therefore consisted of 7,778 leaders rated by 22,997 subordinates (*M* = 2.96, *SD* = 1.28, range 1–9) and 10,132 superiors (*M* = 1.30, *SD* = 0.57, range = 1–6), operating across 53 different industries, including banking, consulting, and telecommunications. The leader sample consisted of 5,958 men (76.60%) and 1,820 women (23.40%) with an average age of 41.16 years (*SD* = 6.23) and 52 different nationalities. The subordinate sample consisted of 14,788 men (64.30%) and 8,209 women (35.70%) with an average age of 39.36 years (*SD* = 8.44) and 56 different nationalities. The superior sample consisted of 8,962 men (88.45%) and 1,170 women (11.55%) with an average age of 48.40 years (*SD* = 7.44) and 54 different nationalities.

In dyadic terms, 16,572 (72.06%) leader-subordinate dyads and 6,630 (65.44%) leader–superior dyads share the same nationality in terms of country of origin. Importantly, although this leaves sizable samples of leader-subordinate and leader-superior dyads that do not share their country of origin, this does not necessarily mean that they are culturally dissimilar, as there may still be substantial overlap in terms of the number of values, beliefs, and norms they share. Previous research has demonstrated that countries can also be pooled into cultural clusters that determine how similar they are culturally (Ronen and Shenkar, 2013). To get a better appreciation of the cultural diversity of leader-observer dyads within our sample, therefore, Table 1 display the distribution of leader-subordinate and leader–superior dyads according to ten cultural clusters previously devised by GLOBE researchers (Gupta et al., 2002), GLOBE being the general theoretical framework we employ. These tables illustrate that 18,502 (80.45%) leader-subordinate dyads and 7,619 (75.20%) leader–superior dyads are from the same cultural cluster. Furthermore, leaders, subordinates, and superiors are represented across all ten clusters, with leader–subordinate dyads representing 90% of possible cluster-combinations and leader-superior dyads 82%. Although certain (combinations of) clusters are more represented than others, the overall distribution within and between cluster combinations appears relatively equal.

**TABLE 1:** Leader–subordinate cultural cluster distribution.

	Subordinate cultural cluster										
	
	1	2	3	4	5	6	7	8	9	10	*Total*
**Leader cultural cluster**											
(1) Anglo	5143	463	47	232	53	254	14	44	239	8	*6497*
(2) Confucian Asia	121	2232	1	23	1	20	1	0	131	2	*2532*
(3) Eastern Europe	31	2	821	25	0	31	1	2	3	1	*917*
(4) Germanic Europe	336	145	82	3864	43	233	21	52	84	2	*4862*
(5) Latin America	19	17	1	21	593	30	3	1	11	0	*696*
(6) Latin Europe	271	186	84	209	98	2850	30	31	87	1	*3847*
(7) Middle East (Arab)	15	3	6	3	5	12	217	0	26	0	*287*
(8) Nordic Europe	101	50	19	53	6	55	10	1274	24	0	*1592*
(9) Southern Asia	82	105	5	17	6	18	5	1	1326	0	*1565*
(10) Sub-Saharan Africa	12	2	0	2	0	1	1	0	2	182	*202*
*Total*	*6131*	*3205*	*1066*	*4449*	*805*	*3504*	*303*	*1405*	*1933*	*196*	*22997*

	**Superior cultural cluster**										
	
	**1**	**2**	**3**	**4**	**5**	**6**	**7**	**8**	**9**	**10**	***Total***

**Leader cultural cluster**											

(1) Anglo	2271	90	7	146	21	116	4	35	30	0	*2720*
(2) Confucian Asia	215	897	0	51	0	40	1	12	46	0	*1262*
(3) Eastern Europe	81	0	240	31	4	53	2	5	2	0	*418*
(4) Germanic Europe	262	13	7	1680	13	96	2	32	9	0	*2114*
(5) Latin America	46	1	0	22	185	21	2	1	3	0	*281*
(6) Latin Europe	255	24	10	165	15	1240	8	27	5	2	*1751*
(7) Middle East (Arab)	26	2	1	8	3	20	88	4	6	0	*158*
(8) Nordic Europe	41	5	3	43	1	15	0	524	1	0	*633*
(9) Southern Asia	162	38	0	39	3	28	0	11	426	1	*708*
(10) Sub-Saharan Africa	11	0	1	1	0	5	0	0	1	68	*87*
*Total*	*3370*	*1070*	*269*	*2186*	*245*	*1634*	*107*	*651*	*529*	*71*	*10132*

### Measures

#### Cultural Distance

Cultural distance reflects the extent to which dyads of individuals are culturally proximal (i.e., a score of 0) or increasingly distal (i.e., scores increasingly higher than 0). Operationalizations of cultural distance may differ depending (1) on the used cultural typology and (2) on the used method of calculation (see Beugelsdijk et al., 2018 for a review). First, cultural distance measures can be based on different typologies of underlying cultural dimensions (Hofstede, 1980, 2001; Schwartz, 1999; House et al., 2004). These typologies differ substantially in content and are typically lowly correlated amongst each other, which makes it important to decide on a typology based on theoretical grounds (Beugelsdijk et al., 2018). Following previous research (e.g., Kossek et al., 2017) and our theoretical approach, we use the GLOBE dimensions as a basis for our cultural distance measure. The GLOBE dimensions are assertiveness, gender egalitarianism, future orientation, humane orientation, in-group collectivism, institutional collectivism, performance orientation, power distance, uncertainty avoidance, and come in both ‘as is’ practices (i.e., the cultural characteristics that leaders actually enact) and ‘should be’ values (i.e., the cultural characteristics that leaders idealize) (House et al., 2004). We use values as they better reflect people’s implicit leadership theories (Dorfman et al., 2012). Second, cultural distance measures can be based on different calculation methods, including Euclidean- and Mahalanobis-based indices. One of the advantages of the Mahalanobis index is that it takes the full variance-co-variance matrix of the underlying dimensions into account (Mahalanobis, 1937), which has a substantial impact on the resulting cultural distance measure when its underlying cultural dimensions are not very highly correlated (Brereton and Lloyd, 2016). Although research typically uses an Euclidean approach (e.g., Kossek et al., 2017), the Mahalanobis approach is more appropriate for our purposes as it corrects for the covariances of the GLOBE dimensions (Beugelsdijk et al., 2018). Accordingly, for our main analyses, we operationalized cultural distance based on a Mahalanobis index of the GLOBE values dimensions, which we derived from Beugelsdijk et al. (2018; see Berry et al., 2010 for more details on how Mahalanobis distance was calculated). We would note, however, that conclusions drawn from our analyses are similar regardless of whether we use a Mahalanobis or Euclidean distance measure.

#### Transformational and Transactional Leadership Behaviors

The Global Executive Leadership Inventory (GELI) is a 360-degree leadership feedback instrument (Kets De Vries et al., 2004). The instrument consists of twelve leadership behaviors and characteristics: visioning, empowering, energizing, designing and controlling, rewarding and feedback, team building, outside orientation, global mindset, tenacity, emotional intelligence, life balance, and resilience to stress. The instrument employs a 7-point Likert scale to indicate how well the scale items describe a leader. The continuum of responses ranges from “does not describe me at all” to “describes me very well.” We used the GELI to operationalize transformational and transactional leadership behaviors. The advantage of using the GELI to examine self-other agreement in transformational and transactional leadership is that its items reflect actual behaviors described and observed in the last two decades in leaders around the globe (Kets De Vries et al., 2004), which forgoes some of the recent criticisms of transformational and transactional leadership operationalizations (e.g., van Knippenberg and Sitkin, 2013).

We operationalized *transformational leadership* using the eight-item visioning dimension of the GELI (“[a]rticulating a compelling vision, mission and strategy with a multi-country, multi-environment, multi-function and multi-gender perspective that connects employees, shareholders, suppliers and customers on a global scale” [Kets De Vries et al., 2004, p. 479]). This dimension aligns with the notion of transformational leadership behaviors as transforming and motivating followers beyond performance expectations by providing a vision, inspiration, and considering employees and other in their unique individuality (cf. Bass and Avolio, 1990) and adheres to the notion that charismatic elements serve as the core of transformational leadership (Shamir et al., 1993; Conger and Kanungo, 1994; van Knippenberg and Sitkin, 2013). An example item includes “I inspire my people to look beyond existing limitations.” The instrument was internally consistent for both leader self-ratings (α = 0.73) and observer other-ratings (α = 0.85).

We operationalized *transactional leadership* using the eight-item rewarding and feedback dimension of the GELI (“[s]etting up the appropriate reward structures and giving constructive feedback to encourage the kind of behavior that is expected from employees” [Kets De Vries et al., 2004, p. 480]). This dimension aligns with the notion of transactional leadership behaviors as motivating followers to meet performance goals set for them and providing resources and rewards for meeting these goals (cf. Bass and Avolio, 1990) and adheres to the notion that contingent reward serves as the core of transactional leadership (Shamir et al., 1993; Conger and Kanungo, 1994; van Knippenberg and Sitkin, 2013). An example item includes “I make sure that people’s achievements are recognized.” The instrument was internally consistent for both leader self-ratings (α = 0.80) and observer other-ratings (α = 0.90).

#### Control Variables

We controlled for industry (Brutus et al., 1998), leader and observer (similarity in) age and gender (Ostroff et al., 2004; Fleenor et al., 2010; Braddy et al., 2020), and hierarchical position (Lee and Carpenter, 2018), as they have been shown to be sources of self-other agreement. Furthermore, given cultural influences on self-other agreement (Atwater et al., 2009; Kossek et al., 2017), we included fixed effects for leader and observer nationality, to ensure that they do not distort our cultural distance effects (Kirkman et al., 2006; Harzing and Pudelko, 2016; Beugelsdijk et al., 2017). Finally, we controlled for the year in which the surveys were administered through dummies to rule out time effects.

### Analyses

To test our two hypotheses, following previous research studying effects on self-other agreement (Ostroff et al., 2004; McKee et al., 2018), we employed the multivariate regression approach to self-other agreement (Edwards, 1995). Within this approach, self-other agreement is estimated through multivariate models in which both self- and other-ratings are simultaneously regressed on the same set of predictors. The effects of a predictor on self- and other-ratings can then be compared to gauge whether this predictor contributes to disagreement. Predictors are considered sources of disagreement when their effects on self- and other-ratings is statistically different. This multivariate approach is superior to alternative approaches such as difference scores, as it allows for identification of where disagreement comes from, in this case the self and/or other (for a review, see Fleenor et al., 2010).

We used Stata (StataCorp, 2017) to estimate generalized structural equation models in which we simultaneously regressed leader- and observer-rated transformational and transactional leadership behaviors on the predictors. We used a cluster-robust estimator (Rogers, 1994) to cluster standard errors at the leader level (*n* = 7,778) to account for the potential bias resulting from leaders being rated by multiple observers (cf. McKee et al., 2018). We chose this approach as previous research has demonstrated that reliabilities within different groups of observers are considered to be relatively low (Conway and Huffcutt, 1997) and because we do not presuppose theoretical differences in within- and between-group estimates (cf. mixed effects models). We standardized the independent variables (i.e., gender, age, hierarchical position, and cultural distance) and used Wald tests to compare the effects of the predictors across the different self- and other-ratings.

## Results

### Descriptive Statistics

Descriptive statistics and inter-correlations can be found in Table 2. Out of the 33,129 leader–observer dyads, 23,202 (70.04%) were culturally proximal (i.e., had a cultural distance of 0) and 9,927 (29.96%) were culturally distal (i.e., had a cultural distance larger than 0). In line with general self-other agreement findings (e.g., Harris and Schaubroeck, 1988; Lee and Carpenter, 2018), correlations between self- and other-ratings of transformational leadership, *r*(33,219) = 0.14, *p* < 0.001, and transactional leadership, *r*(33,219) = 0.15, *p* < 0.001, were relatively low. Furthermore, in line with previous research (for an overview, see Fleenor et al., 2010), older, *r*(33,219) = 0.06, *p* < 0.001, and male leaders *r*(33,219) = –0.02, *p* < 0.001, provided higher self-ratings on transformational leadership than younger and female leaders. Finally, correlation magnitudes are roughly in line with previous research (Ostroff et al., 2004).

**TABLE 2:** Descriptive statistics and intercorrelations for leader–observer dyads.

	*M*	*SD*	*Min*	*Max*	1	2	3	4	5	6	7	8	9
(1) Age (leader)	41.30	6.16	24.00	77.00	–								
(2) Age (observer)	42.12	9.15	22.00	81.00	0.27***	–							
(3) Gender (leader)	0.22	0.42	0.00	1.00	–0.02***	–0.04***	–						
(4) Gender (observer)	0.28	0.45	0.00	1.00	–0.04***	–0.23***	0.16***	–					
(5) Cultural distance (leader vs. observer)	4.17	7.82	0.00	55.38	–0.04***	–0.02**	0.01	0.01*	–				
(6) Transformational leadership (leader)	5.73	0.58	2.50	7.00	0.09***	0.03***	–0.03***	0.00	0.03***	(0.73)			
(7) Transformational leadership (observer)	5.53	0.84	1.00	7.00	0.01	–0.04***	0.00	0.06***	–0.01	0.14***	(0.85)		
(8) Transactional leadership (leader)	5.52	0.70	1.75	7.00	0.06***	–0.01	0.05***	0.00	0.04***	0.50***	0.09***	(0.80)	
(9) Transactional leadership (observer)	5.27	1.00	1.00	7.00	0.01*	0.03***	0.05***	–0.02**	0.02***	0.07***	0.64***	0.15***	(0.90)

*N = 33,219. Gender is coded as 0 = male, 1 = female. Cronbach’s Alpha’s on the diagonal.*

*p < 0.05, **p < 0.01, ***p < 0.001.

### Hypotheses Testing

Hypothesis 1 stated that cultural distance is negatively related to self-other agreement. To test this hypothesis, we estimated a model in which we used all control variables and cultural distance as predictors of transformational and transactional leadership (see Table 3). First, in terms of transformational leadership, results indicate that cultural distance is positively related to self-ratings of transformational leadership (*b* = 0.011, *p* < 0.05, column 1), and negatively related to other-ratings of transformational leadership, (*b* = –0.020, *p* < 0.001, column 2). The Wald test reveals that the effect of cultural distance on self- and other-ratings of transformational leadership are significantly different [*b* = 0.032, χ*2*(1) = 21.93, *p* < 0.001, column 3]. Second, for transactional leadership, results indicate that cultural distance is positively related to self-ratings (*b* = 0.012, *p* < 0.10, column 4), and negatively related to other-ratings of transactional leadership (*b* = –0.012, *p* < 0.10, column 5) at a marginal significance level. The Wald test reveals that the effects of cultural distance on self- and other-ratings of transactional leadership are significantly different [*b* = 0.024, χ*2*(1) = 8.00, *p* < 0.01, column 6]. Accordingly, these results provide support for Hypothesis 1, by demonstrating that cultural distance is negatively associated with self-other agreement. Interestingly, for both transformational and transactional leadership, this association with self-other agreement is determined both by increased self-ratings and decreased other-ratings.

**TABLE 3:** Multivariate regressions and Wald tests for leader-observer dyads.

	Transformational leadership			Transactional leadership		
		
	Leader	Observer	Disagreement	Leader	Observer	Disagreement
Constant	5.825***	5.665***	0.160	6.118***	5.709***	0.409
	(0.259)	(0.453)	(0.292)	(0.297)	(0.384)	(0.343)
Age (leader)	0.045***	–0.022***	0.066***	0.054***	0.006	0.049***
	(0.007)	(0.006)	(0.009)	(0.008)	(0.008)	(0.010)
Age (observer)	0.014**	0.032***	–0.018*	–0.003	0.016*	–0.019*
	(0.005)	(0.006)	(0.007)	(0.006)	(0.007)	(0.008)
Age (leader) × Age (observer)	0.001	0.000	0.001	–0.003	0.008	–0.011
	(0.004)	(0.005)	(0.006)	(0.005)	(0.006)	(0.007)
Gender (leader)	–0.023**	–0.010	–0.013	0.028***	0.031***	–0.003
	(0.007)	(0.006)	(0.008)	(0.008)	(0.007)	(0.010)
Gender (observer)	0.000	0.021***	–0.021**	–0.005	–0.009	0.004
	(0.004)	(0.005)	(0.006)	(0.004)	(0.007)	(0.007)
Gender (leader) × Gender (observer)	–0.001	0.006	–0.008	0.003	0.010^†^	–0.007
	(0.003)	(0.005)	(0.005)	(0.004)	(0.006)	(0.006)
Hierarchical position (subordinate vs. superior)	–0.006^†^	–0.105***	0.099***	0.002	0.083***	–0.081***
	(0.003)	(0.005)	(0.006)	(0.004)	(0.006)	(0.007)
Cultural distance (leader–observer)	0.011*	–0.020***	0.032***	0.012^†^	–0.012^†^	0.024**
	(0.005)	(0.006)	(0.007)	(0.006)	(0.007)	(0.008)
*R* ^2^	0.053	0.049		0.080	0.050	
*Adjusted R^2^*	0.048	0.044		0.075	0.045	

*N = 33,129. Standard errors clustered at leader level (*N* = 7,778). Fixed effects for year, industry, nationality (leader), and nationality (observer) are included but not reported. Standard errors between parentheses.*

^†^*p* < 0.10, *p < 0.05, **p < 0.01, ***p < 0.001.

Hypothesis 2 stated that the effect of cultural distance on self-other agreement is moderated by the hierarchical position of the observer (i.e., subordinate vs. superior), such that the relationship is stronger for superiors than for subordinates. To test this hypothesis, we reran the previous model (see Table 3) with the interaction term between hierarchical position and cultural distance added as predictor of transformational and transactional leadership self- and other-ratings (see Table 4), and we calculated simple main effects (see Table 5). First, for transformational leadership, results in Table 4 indicate that the interaction is unrelated to self-ratings (*b* = 0.004, *ns*), and negatively to other-ratings (*b* = –0.011, *p* < 0.05). The Wald test reveals that the interaction effects are significantly different for self- and other-ratings [*b* = 0.014, χ*2*(1) = 6.53, *p* < 0.05]. As Table 5 shows, simple main effects of cultural distance on self-other agreement on transformational leadership for leader-subordinate dyads (*b* = 0.021, *p* < 0.05), and leader-superior dyads (*b* = 0.053, *p* < 0.001), are indeed significantly different [*b* = –0.031, χ*2*(1) = 6.53, *p* < 0.05]. Second, for transactional leadership, results in Table 4 indicate that the interaction is unrelated to self-ratings, (*b* = 0.005, *ns)*, and negatively to other-ratings (*b* = –0.011, *p* < 0.05). The Wald test reveals that the interaction effects are significantly different for self- and other-ratings [*b* = 0.016, χ*2*(1) = 6.05, *p* < 0.05]. As Table 5 shows, simple main effects of cultural distance on self-other agreement on transactional leadership for leader-subordinate dyads (*b* = 0.012, *ns*), and leader-superior dyads (*b* = 0.046, *p* < 0.001), are significantly different [*b* = –0.034, χ*2*(1) = 6.05, *p* < 0.05]. Taken together, these results provide support for Hypothesis 2 by demonstrating that the negative relationship between cultural distance and self-other agreement is stronger for leader-superior than for leader-observer dyads. Interestingly, for both transformational and transactional leadership, this result is determined primarily by lower other-ratings.

**TABLE 4:** Multivariate regressions and Wald tests for leader-observer dyads.

	Transformational leadership			Transactional leadership		
		
	Leader	Observer	Disagreement	Leader	Observer	Disagreement
Constant	5.826***	5.661***	0.165	6.119***	5.705***	0.415
	(0.259)	(0.451)	(0.289)	(0.297)	(0.382)	(0.342)
Age (leader)	0.044***	–0.021***	0.066***	0.054***	0.006	0.048***
	(0.007)	(0.006)	(0.009)	(0.008)	(0.008)	(0.010)
Age (observer)	0.014**	0.032***	–0.018*	–0.003	0.015*	–0.019*
	(0.005)	(0.006)	(0.007)	(0.006)	(0.007)	(0.008)
Age (leader) × Age (observer)	0.001	–0.000	0.001	–0.003	0.007	–0.010
	(0.004)	(0.005)	(0.006)	(0.005)	(0.006)	(0.007)
Gender (leader)	–0.023**	–0.010	–0.014	0.028***	0.031***	–0.004
	(0.007)	(0.006)	(0.008)	(0.008)	(0.007)	(0.010)
Gender (observer)	0.000	0.021***	–0.021**	–0.005	–0.009	0.004
	(0.004)	(0.005)	(0.006)	(0.004)	(0.007)	(0.007)
Gender (leader) × Gender (observer)	–0.001	0.006	–0.008	0.003	0.010^†^	–0.007
	(0.003)	(0.005)	(0.005)	(0.004)	(0.006)	(0.006)
Hierarchical position (subordinate vs. superior)	–0.006^†^	–0.105***	0.099***	0.002	0.083***	–0.081***
	(0.003)	(0.005)	(0.006)	(0.004)	(0.006)	(0.007)
Cultural distance (leader-observer)	0.011*	–0.020***	0.031***	0.012^†^	–0.011	0.023**
	(0.005)	(0.006)	(0.007)	(0.006)	(0.007)	(0.009)
Cultural distance × Hierarchical position	0.004	–0.011*	0.014*	0.005	–0.011*	0.016*
	(0.003)	(0.005)	(0.006)	(0.004)	(0.006)	(0.006)
*R* ^2^	0.053	0.050		0.080	0.050	
*Adjusted R^2^*	0.048	0.045		0.075	0.045	

*N = 33,129. Standard errors clustered at leader level (*N* = 7,778). Fixed effects for year, industry, nationality (leader), and nationality (observer) are included but not reported. Standard errors between parentheses.*

^†^p < 0.10, *p < 0.05, **p < 0.01, ***p < 0.001.

**TABLE 5:** Simple main effects for leader-observer dyads.

Hierarchical position	Leadership behavior					
	
	Transformational leadership			Transactional leadership		
		
	Leader	Observer	Disagreement^b^	Leader	Observer	Disagreement^b^
Subordinate	0.009	–0.012^†^	0.021*	0.009	–0.004	0.012
	(0.006)	(0.007)	(0.008)	(0.007)	(0.009)	(0.011)
Superior	0.017**	–0.036***	0.053***	0.019*	–0.028**	0.046***
	(0.006)	(0.009)	(0.010)	(0.008)	(0.009)	(0.011)
Disagreement^a^	–0.008	0.023*	–0.031*	–0.010	0.024*	–0.034*
	(0.007)	(0.011)	(0.012)	(0.009)	(0.012)	(0.014)

*N = 33,129. Standard errors clustered at leader level (*N* = 7,778). Standard errors between parentheses.*

*^*a*^Wald tests comparing simple main effects of Subordinate vs. Superior within Leader or Observer, ^*b*^Wald tests comparing Leader vs. Observer within Subordinate or Superior.*

^†^*p* < 0.10, *p < 0.05, **p < 0.01, ***p < 0.001.

### Robustness Checks

To check the robustness of our findings, we conducted several additional analyses. First, acknowledging that the number of culturally similar leader-observer dyads in our sample is relatively high (70.04%), we alternated the ratios of culturally proximal vs. culturally distal dyads (60% vs. 40%, 50% vs. 50%, 40% vs. 60%, and 30% vs. 70%) by drawing random subsamples of culturally similar leader-observer dyads. We reran our main analyses using these random subsamples, of which results are reported in Appendices A1, A2. These results are very comparable to those from our main analyses. That is, except for a marginally significant interaction effect on self-other agreement of transactional leadership for the 30% vs. 70% subsample, cultural distance effects were significant across all subsamples. Our conclusions are therefore relatively robust against overrepresentation of culturally proximal dyads and underrepresentation of culturally distal dyads.

Second, acknowledging the different approaches to operationalizing cultural distance, we reran our analyses using seven alternative Mahalanobis-based cultural distance measures (see also Beugelsdijk et al., 2018): (a) GLOBE practices, (b) Hofstede (2001), (c) Schwartz (1999), (d) Berry et al. (2010), (e) Beugelsdijk et al. (2015a), (f) a composite of the Hofstede and Schwartz dimensions, and (g) a composite of the GLOBE values, Hofstede, and Schwartz dimensions, of which results are reported in Appendices B1, B2. Results from these supplementary analyses are very comparable to those from our main analyses. That is, except for a non-significant interaction effect on self-other agreement of transformational leadership for the Beugelsdijk et al. (2015a) measure, cultural distance effects were significant across all cultural distance measures. Our conclusions are therefore relatively robust for different cultural distance measures.

## Discussion and Implications

In this paper, we analyzed how multisource feedback operates in a multicultural context. Specifically, we studied how cultural distance between leaders and observers relates to self-other agreement. First, drawing from leadership categorization theory (Lord et al., 1982, 1984; Lord and Maher, 1991), we hypothesized and found that cultural distance is associated with lower self-other agreement on transformational and transactional leadership behaviors. Results indicate that cultural distance negatively relates to self-other agreement in two ways. First, cultural distance negatively relates to the other-ratings provided by observers. This finding is in line with leadership categorization theory (Lord et al., 1982, 1984; Lord and Maher, 1991) and research on implicit leadership theories in intercultural settings (House et al., 2004). Moreover, we also find that cultural distance relates to higher self-ratings provided by leaders. One possible explanation for the latter finding might lie in the fact that, in general, inflated self-perceptions of leadership behaviors like transformational behavior boost leaders’ feelings of efficacy and confidence (Taylor and Brown, 1988, 1994). It is conceivable that in a context with culturally distal superiors or subordinates, feeling confident is even more important, such that the inclination to self-enhance as a leader is stronger. This may hold especially true for bicultural leaders that may additionally rely on their cross-cultural competences and multiple cultural profiles (Lakshman, 2013).

Second, drawing upon the approach/inhibition theory of power (Keltner et al., 2003), we hypothesized and found that the negative relationship between cultural distance and self-other agreement depends on the hierarchical position of the observer. Results consistently show that the effects of cultural distance are indeed stronger for leader-superior than for leader-subordinate dyads. This dissimilarity is primarily determined by lower scores of superiors. In line with findings of Galinsky et al. (2006), our results seem to support the idea that people with high power are less dependent on others, and therefore it is less important for them to have an accurate and comprehensive understanding of others, in our case their leaders.

### Theoretical Implications for Self-Other Agreement in Multisource Feedback

To the best of our knowledge, our study is the first to investigate the role of cultural distance in multisource feedback and self-other agreement. Whereas previous research has shown that self-other agreement operates differently across countries and cultures (e.g., Atwater et al., 2005; Gentry et al., 2007; Varela and Premeaux, 2008) and that being from a different culture or country can influence evaluations (e.g., Caligiuri and Day, 2000; Testa, 2002, 2009; Smith et al., 2012; Kossek et al., 2017), no study known to us has studied how cultural *distance* influences self-other *agreement*, especially not when simultaneously considering the hierarchical position of observers. By doing so, the current study integrates research on self-other agreement with leadership categorization theory (Lord et al., 1982, 1984; Lord and Maher, 1991) and the approach/inhibition theory of power (Keltner et al., 2003). Previous research has come up with various cognitive mechanisms to explain self-other agreement (e.g., McKee et al., 2018), including topics as observability and criteria used when providing self- and other-ratings (Tsui and Ohlott, 1988; Yammarino and Atwater, 1993; Oh and Berry, 2009). Our integrated theoretical perspective and the findings of our study have three theoretical implications.

First, our study has implications for the interpretation and meaning of leader- and observer-ratings in self-other agreement and multisource feedback. Notwithstanding some exceptions (e.g., Fleenor et al., 2010; McKee et al., 2018), the self-other agreement literature in general assumes that self-ratings are considered to be less accurate than other-ratings (e.g., Atwater et al., 1998). Self-other agreement is often assumed to indicate self-awareness on the leaders’ part; the dominant assumption is that disagreement primarily indicates that *leaders* over- or underestimate themselves. This perspective ignores the fact that leaders’ ratings may be accurate and that *observers* over- or underestimate the behaviors of the leader (cf. Van der Kam et al., 2014; McKee et al., 2018). Our findings again confirm the limitations of this perspective, because we demonstrate that observer ratings can serve as the primary source of self-other agreement, when cultural distance is taken into account. Moreover, the fact that the relevance of cultural distance is conditional upon the hierarchical position of the observer, serves to exuberate the idea that self-other agreement is driven more strongly by observers’ ratings, particularly in case of superior ratings. Given the overwhelming evidence for biases in self-ratings (Harris and Schaubroeck, 1988; Tsui and Ohlott, 1988; Yammarino and Atwater, 1993; Atwater et al., 1998), we would of course not claim that leaders’ self-ratings are by definition less biased than observers’ ratings. Indeed, to be able to answer whether self- and/or other-ratings are biased, one would need to apply a componential approach using round-robin data (Kwan et al., 2004; Van der Kam et al., 2015), which our dataset unfortunately did not allow for. Notwithstanding this limitation, our results suggest that the field of organizational psychology should further acknowledge that leaders’ self-ratings are not necessarily more (in)accurate than observers’ ratings, especially in case of cultural distance between the leader and the observer, and especially when it concerns ratings of the superior.

Second, extending the previous point, our study has implications for the role of self-awareness in the self-other agreement literature. The self-other agreement literature strongly assumes that classifying leaders as self-aware individuals, overestimators, or underestimators, provides meaningful directions for leader development (Yammarino and Atwater, 1993; Atwater et al., 1998). These classifications are based on an assumption that leaders and observers use equivalent criteria, and also on the assumption that observers’ ratings are more accurate than those by leaders themselves. Our findings suggest that leaders and observers with distinct cultural backgrounds probably have different implicit leadership theories which influence their expectations about appropriate leadership and therefore also influence the criteria they use to assess leadership behaviors (Tsui and Ohlott, 1988; Yammarino and Atwater, 1993; Oh and Berry, 2009).

Third, our study has implications for self-other agreement of transformational and transactional leadership behaviors. Transformational and transactional leadership behaviors are often studied within the context of self-other agreement (e.g., Bass and Yammarino, 1991; Whittington et al., 2009; Ertürk et al., 2018; Vogel and Kroll, 2019). Meta-analytic evidence suggests that leaders and observers are more likely to disagree on transformational than on transactional leadership (Lee and Carpenter, 2018). Although social desirability effects have been suggested to be a source of these differences, it is not clear whether this is actually the case. Furthermore, notwithstanding specific boundary conditions (Crede et al., 2019), there is mixed empirical evidence as to whether such leadership behaviors are culturally universal (Den Hartog et al., 1999) or whether there are specific cultural elements to them (e.g., Gerstner and Day, 1994; Dorfman et al., 1997; Money and Graham, 1999; Javidan and Carl, 2004; Karakitapoglu-Aygün and Gumusluoglu, 2013). Our study demonstrates that cultural distance does influence self-other agreement on both transformational and transactional leadership behaviors – more so for leader–superior than for leader-subordinate dyads.

Finally, notwithstanding slight differences in effect sizes, the relationships between cultural distance and self-other agreement of transformational and transactional leadership are relatively homogeneous across the two leadership behaviors. This result has important theoretical implications for where we can attribute the source of variance to. Specifically, in our study, cultural distance is both (marginally) positively related to self-ratings and (marginally) negatively related to other-ratings, with the effect of superior ratings being even stronger than subordinate ratings. This implies that the rating sources (i.e., self vs. subordinate vs. superior) are not necessarily interchangeable and that these sources may have different conceptualizations of leadership (cf. Lee and Carpenter, 2018). Given the consistent outcomes across transformational and transactional leadership in our study, therefore, it seems plausible to conclude that cultural distance elicits more general cognitive biases effects rather than effects tied into specific leadership styles. On the one hand, these results contribute to understanding some of the unexplained heterogeneity in self-other agreement differences between transformational and transactional leadership (Lee and Carpenter, 2018). On the other hand, these outcomes contribute to the debate about the cultural universality or specificity of transformational and transactional by suggesting that leaders and observers may hold culturally dependent implicit leadership theories about these behaviors.

### Practical Implications for Self-Other Agreement in Culturally Dissimilar Dyads

One of the strengths of the self-other agreement literature is that it has immense implications for various groups of practitioners, including the leaders being rated, the observers providing the ratings, and the HR-managers responsible for the multisource feedback systems that facilitate these ratings. The results of our study introduce various relevant practical implications affecting all of these groups, but especially for HR-managers. First and foremost, our study suggests a clear bias of observers against culturally distant leaders. Because culturally distant leaders are already faced with various challenges (Ocker et al., 2011; Kossek et al., 2017), to avoid invalid and even unfair evaluations of their leader behaviors, HR-professionals should use and interpret self-other agreement across culturally different leader–observer dyads with caution.

The bias in self-other agreement seems to originate from different lenses or criteria when raters rate leaders’ behaviors (Tsui and Ohlott, 1988; Yammarino and Atwater, 1993; Oh and Berry, 2009). The implicit leadership theories we hold are not the same across all cultures and contexts (Gerstner and Day, 1994; Den Hartog et al., 1999; House et al., 2004; Dorfman et al., 2012), and these theories affect how we rate culturally similar and dissimilar others (Shaw, 1990; Newman and Nollen, 1996). It is important for both leaders and observers to be aware of this fact (Lu and Wan, 2018). More generally, organizations can reduce the effects of this bias, for instance, by explicitly and specifically stating which aspects of leadership behaviors are to be rated, or by increasing cultural awareness through intercultural sensitivity training programs (Landis and Bhagat, 1996; Lu and Wan, 2018).

Third, self-other agreement is often used as input to make crucial HR-decisions with respect to leader development and performance, and sometimes even promotion (Ostroff et al., 2004; Fleenor et al., 2010). The reliability and viability of these decisions and outcomes critically hinges upon both self- and other-ratings being void of bias. Our results show that in situations of cultural distance, such ratings have to be used with caution, especially the ratings by superiors. If all parties involved really want to use multisource feedback systems for both culturally similar and culturally similar leader–observer dyads, it is imperative that they acknowledge how cultural distance can influence the rating process, and especially how ratings by culturally different superiors are driven by implicit leadership theories.

### Limitations and Future Research Directions

Notwithstanding its merits, our study is not without limitations. There are several methodological and statistical issues that limit our contribution. First, we used a cross-sectional design in which we administered the survey to measure our variables at the same time. To be able to make causal claims, a preferred research design would include repeated measures of the same leaders with multiple ratings of culturally similar *and* dissimilar observers. Second, while the instruments were administered to a wide variety of leaders across various contexts, industries, and countries allowing for broad generalizations, most of the leaders were relatively high up the hierarchical level. Although research has demonstrated that leader status is unrelated to self-other agreement (Lee and Carpenter, 2018), this cannot rule out that the effects demonstrated in our study do not apply to leaders lower down the hierarchical ladder. Third, leaders were personally responsible for nominating observers that would provide honest feedback. Given the instructions provided to the leaders and the leadership development program they were in, we are relatively confident that they would draw a representative sample of observers. We cannot rule out, however, the potential for selection bias that could affect the reliability of the other-ratings. Fourth, the data we used in our study were collected between 2003 and 2012, which makes the dataset nearly a decade old at the time of writing. Notwithstanding the fact that our results are robust to temporal effects in the decade under study (i.e., we controlled for year in our estimations), our results may be subject to other temporal effects occurring after the period our data were gathered. Specifically, while the relative distances between cultures are likely to remain stable over time (Beugelsdijk et al., 2015b), people’s cognitive schemas about how to handle cultural distance in terms of receiving and providing feedback may be subject to temporal changes.

Fifth, because we had no information about the organization of the participants in the leadership development program, we were unable to control for the actual geographical location of the organization. Accordingly, although we know when leaders and observers originate from different countries, we do not know which one can be considered the expatriate. This is unfortunate, as expatriates are typically treated differently than locals (Kossek et al., 2017). Hence, knowing whether a leader is an expatriate or a local may have important implications for the type of leadership behaviors they prefer and how they are accordingly are perceived by observers. In a similar vein, we used cultural values at the country-level as input for our cultural distance measure, which may not represent the personal values at the individual level. Although cultural and personal values are likely to be related (cf. the cultural expertise and personal values proposition, Peterson and Barreto, 2014), these studying these cultural values at the country-level may have attenuated our findings. Moreover, in line with our theoretical approach, we used a cultural distance measure based on a configuration of cultural dimensions rather than studying single cultural value dimensions. While this approach corresponds with previous cultural distance research (Beugelsdijk et al., 2018), this does not rule out that distance on some cultural dimensions may have stronger effects on self-other agreement than others. Although we have explored the effects of cultural distance on specific cultural dimensions (results available on request from the first author), found that certain cultural dimensions had stronger effects than others, we were unable to draw meaningful conclusions within the scope of this manuscript. Still, disentangling distance effects of specific cultural dimensions could increase our understanding of how self-other agreement operates in intercultural contexts. Similarly, although we have focused on studying culture, there are several other societal and national factors that could contribute to self-other agreement in multinational teams, including differences in development, language, or status (e.g., Haas, 2005; Neeley, 2013; Paunova, 2017).^1^

Also, we do not explicitly test whether the relationship between cultural distance and self-other agreement is indeed driven by implicit leadership theories. Recent studies have demonstrated that culturally endorsed leadership attributes serve as crucial mediating mechanisms between (leadership) variables that are seemingly unrelated (Dorfman et al., 2012). Although measuring implicit leadership theories is no precondition for using leadership categorization theory as an explanatory framework (e.g., Den Hartog et al., 1999; House et al., 2004), not measuring them opens up the possibility that our effects could be driven by alternative (causal) mechanisms. Indeed, whereas we assume that leader prototypes influence leader evaluations, earlier research on leadership categorization theory suggest that leader evaluations influence leader prototypes (e.g., Phillips and Lord, 1982; Cronshaw and Lord, 1987). This seems unlikely within our study context given that implicit leadership theories are culturally dependent (Gerstner and Day, 1994; Den Hartog et al., 1999; House et al., 2004), but is not something we can rule out at this time. In a similar vein, our results could instead be driven by in-group favoritism effects due to similarity-attraction mechanisms (Byrne et al., 1986). Given that we find no such similarity-attraction effects for age and gender, however, leads us to believe that our effects are unlikely to be driven merely by similarity-attraction mechanisms. Still, there are various other characteristics, such as race and personality, not available in our data that may yet influence self-other agreement through similarity-attraction processes (Antonioni and Park, 2001; Ostroff et al., 2004; Rosette et al., 2008). Future research could more explicitly test whether the effects reported here are driven by implicit leadership theories, similarity-attraction concerns, or other theoretically meaningful mechanisms.

Finally, we limited ourselves to studying transformational and transactional leadership behaviors. Although these leadership styles have previously been studied within self-other agreement contexts (e.g., Bass and Yammarino, 1991; Whittington et al., 2009; Ertürk et al., 2018; Vogel and Kroll, 2019), effects for self-other agreement may be different for other leadership styles (Lee and Carpenter, 2018). Furthermore, we used the GELI (Kets De Vries et al., 2004) to operationalize transformational and transactional leadership behaviors, which may not fully capture all underlying dimensions of transformational and transactional leadership. To further explore whether all or specific dimensions of transactional and transformational leadership are culturally contingent (Den Hartog et al., 1999), future research could use more extensive scales (e.g., the MLQ, Bass and Avolio, 1990).

## Conclusion

In our increasingly globalized world, multisource feedback plays a crucial role in guiding leader development (Atwater et al., 1998, 2009; Fleenor et al., 2010; Kossek et al., 2017). An important result of such multisource feedback is self-other agreement, which has shown to be relevant for leader effectiveness (Yammarino and Atwater, 1993; Atwater et al., 1998). Our study suggests that the cultural distance between leaders and observers is a crucial variable for self-other agreement, such that cultural distance is associated with lower self-other agreement on transformational and transactional leadership behaviors. Although it may be tempting to conclude that this disagreement may stem from the culturally distant *leaders*, our results indicate that this disagreement mostly stems from *observers*, and particularly from superiors. It may therefore be inappropriate and even dangerous to base important development and promotion decisions on such potentially biased self-other agreement within intercultural contexts. Given the importance and prevalence of multisource feedback in gauging leader effectiveness and development (Yammarino and Atwater, 1993; Atwater et al., 1998), organizations should interpret the results from multisource feedback systems in culturally dissimilar dyads with caution, by being aware that the ratings are strongly related to the cultural distance between a leader and the observer.

## Data Availability Statement

The data analyzed in this study is subject to the following licenses/restrictions: The data are owned by the Kets de Vries Institute and INSEAD. The authors have no rights to share the data with others. Requests to access these datasets should be directed to MK, research@kdvi.com.

## Ethics Statement

Ethical review and approval was not required for the study on human participants in accordance with the local legislation and institutional requirements. The patients/participants provided their written informed consent to participate in this study.

## Author Contributions

TV, CR, HG, and JS contributed to developing the research idea, conceptualizing the framework, and writing the manuscript. TV contributed to the statistical analyses. CR and MK contributed to data collection. All authors read and approved the submitted version.

## Conflict of Interest

The authors declare that the research was conducted in the absence of any commercial or financial relationships that could be construed as a potential conflict of interest.

## Publisher’s Note

All claims expressed in this article are solely those of the authors and do not necessarily represent those of their affiliated organizations, or those of the publisher, the editors and the reviewers. Any product that may be evaluated in this article, or claim that may be made by its manufacturer, is not guaranteed or endorsed by the publisher.

## Footnotes

1 We thank the reviewer for offering this suggestion.

## Appendix

**Appendix A1:** Multivariate regressions and Wald tests for leader–observer dyads for alternative random samples.

	Transformational leadership			Transactional leadership		
		
	Leader	Observer	Disagreement	Leader	Observer	Disagreement
**70.04% same nationality (*N*_same_ = 23,202) vs. 29.96% other nationality (*N*_other_ = 9,927)**						
Cultural distance (leader–observer)	0.011*	–0.020***	0.032***	0.012 +	–0.012 +	0.024***
	(0.005)	(0.006)	(0.007)	(0.006)	(0.007)	(0.008)
**60% same nationality (*N*_same_ = 14,891) vs. 40% other nationality (*N*_other_ = 9,927)**						
Cultural distance (leader–observer)	0.013*	–0.022**	0.035***	0.012^†^	–0.011	0.023*
	(0.006)	(0.006)	(0.008)	(0.007)	(0.008)	(0.010)
**50% same nationality (*N*_same_ = 9,927) vs. 50% other nationality (*N*_other_ = 9,927)**						
Cultural distance (leader–observer)	0.016*	–0.021**	0.037***	0.013^†^	–0.013	0.026*
	(0.006)	(0.007)	(0.009)	(0.008)	(0.009)	(0.011)
**40% same nationality (*N*_same_ = 6,619) vs. 60% other nationality (*N*_other_ = 9,927)**						
Cultural distance (leader–observer)	0.014*	–0.034***	0.048***	0.017^†^	–0.019*	0.036**
	(0.007)	(0.008)	(0.010)	(0.009)	(0.010)	(0.012)
**30% same nationality (*N*_same_ = 4,255) vs. 70% other nationality (*N*_other_ = 9,927)**						
Cultural distance (leader–observer)	0.016*	–0.025**	0.041***	0.015	–0.010	0.025^†^
	(0.007)	(0.009)	(0.011)	(0.009)	(0.011)	(0.013)

*Standard errors clustered at leader level. Fixed effects for constant, year, industry, nationality (leader), nationality (observer), age (leader), age (observer), age (leader) × age (observer), gender (leader), gender (observer), gender (leader) × gender (observer), and hierarchical position (subordinate vs. superior) are included but not reported. Standard errors between parentheses.*

*^†^*p* < 0.10, **p* < 0.05, ***p* < 0.01, ****p* < 0.001.*

**Appendix A2:** Multivariate regressions and Wald tests for leader–observer dyads for alternative random samples.

	Transformational leadership			Transactional leadership		
		
	Leader	Observer	Disagreement	Leader	Observer	Disagreement
**70% same nationality (*N*_same_ = 23,202) vs. 30% other nationality (*N*_other_ = 9,927)**						
Cultural distance × Hierarchical position	0.004	–0.011*	0.014*	0.005	–0.011*	0.016*
	(0.003)	(0.005)	(0.006)	(0.004)	(0.006)	(0.006)
**60% same nationality (*N*_same_ = 14,891) vs. 40% other nationality (*N*_other_ = 9,927)**						
Cultural distance × Hierarchical position	0.005	–0.012*	0.018**	0.007	–0.012^†^	0.019**
	(0.004)	(0.006)	(0.007)	(0.005)	(0.006)	(0.007)
**50% same nationality (*N*_same_ = 9,927) vs. 50% other nationality (*N*_other_ = 9,927)**						
Cultural distance × Hierarchical position	0.005	–0.014*	0.019*	0.005	–0.013^†^	0.018*
	(0.004)	(0.006)	(0.007)	(0.005)	(0.007)	(0.008)
**40% same nationality (*N*_same_ = 6,619) vs. 60% other nationality (*N*_other_ = 9,927)**						
Cultural distance × Hierarchical position	–0.000	–0.017*	0.017*	0.005	–0.020**	0.025**
	(0.005)	(0.007)	(0.008)	(0.006)	(0.008)	(0.009)
**30% same nationality (*N*_same_ = 4,255) vs. 70% other nationality (*N*_other_ = 9,927)**						
Cultural distance × Hierarchical position	0.003	–0.018*	0.021*	0.002	–0.022**	0.024*
	(0.005)	(0.008)	(0.009)	(0.006)	(0.008)	(0.010)

*Standard errors clustered at leader level. Fixed effects for constant, year, industry, nationality (leader), nationality (observer), age (leader), age (observer), age (leader) × age (observer), gender (leader), gender (observer), gender (leader) × gender (observer), hierarchical position (subordinate vs. superior), and cultural distance (leader–observer) are included but not reported. Standard errors between parentheses.*

*^†^ p < 0.10, * p < 0.05, ** p < 0.01, *** p < 0.001.*

**Appendix B1:** Multivariate regressions and Wald tests for leader–observer dyads for alternative cultural distance measures.

	Transformational leadership			Transactional leadership		
		
	Leader	Observer	Disagreement	Leader	Observer	Disagreement
**GLOBE practices (*N*_dyads_ = 33,129; *N*_leaders_ = 7,778)**						
Cultural distance (leader–observer)	0.012*	–0.020***	0.032***	0.007	–0.015*	0.022**
	(0.005)	(0.006)	(0.007)	(0.006)	(0.006)	(0.008)
**Hofstede (*N*_dyads_ = 32,381; *N*_leaders_ = 7,604)**						
Cultural distance (leader–observer)	0.009^†^	–0.021***	0.030***	0.010^†^	–0.012^†^	0.022**
	(0.005)	(0.006)	(0.007)	(0.006)	(0.006)	(0.008)
**Schwartz (*N*_dyads_ = 32,381; *N*_leaders_ = 7,702)**						
Cultural distance (leader–observer)	0.006	–0.018**	0.023**	0.006	–0.010	0.017*
	(0.005)	(0.005)	(0.007)	(0.006)	(0.007)	(0.008)
**Berry (*N*_dyads_ = 32,082; *N*_leaders_ = 7,547)**						
Cultural distance (leader–observer)	0.006	–0.027***	0.032***	0.008	–0.015*	0.023**
	(0.005)	(0.006)	(0.007)	(0.006)	(0.007)	(0.008)
**Beugelsdijk (*N*_dyads_ = 28,428; *N*_leaders_ = 6,750)**						
Cultural distance (leader–observer)	0.014**	–0.019**	0.032***	0.015*	–0.015*	0.030**
	(0.005)	(0.006)	(0.007)	(0.006)	(0.007)	(0.009)
**Hofstede and Schwartz (*N*_dyads_ = 32,232; *N*_leaders_ = 7,574)**						
Cultural distance (leader–observer)	0.010*	–0.018**	0.028***	0.010	–0.011	0.021*
	(0.005)	(0.005)	(0.007)	(0.006)	(0.007)	(0.008)
**GLOBE, Hofstede, and Schwartz (*N*_dyads_ = 32,232; *N*_leaders_ = 7,574)**						
Cultural distance (leader–observer)	0.008	–0.019***	0.028***	0.010	–0.012^†^	0.022*
	(0.005)	(0.005)	(0.007)	(0.006)	(0.007)	(0.008)

*Standard errors clustered at leader level. Fixed effects for constant, year, industry, nationality (leader), nationality (observer), age (leader), age (observer), age (leader) × age (observer), gender (leader), gender (observer), gender (leader) × gender (observer), and hierarchical position (subordinate vs. superior) are included but not reported. Standard errors between parentheses. ^†^*p* < 0.10, **p* < 0.05, ***p* < 0.01, ****p* < 0.001.*

**Appendix B2:** Multivariate regressions and Wald tests for leader–observer dyads for alternative cultural distance measures.

	Transformational leadership			Transactional leadership		
		
	Leader	Observer	Disagreement	Leader	Observer	Disagreement
**GLOBE practices (*N*_dyads_ = 33,129; *N*_leaders_ = 7,778)**						
Cultural distance × Hierarchical position	0.005	–0.008	0.012*	0.005	–0.010^†^	0.015*
	(0.003)	(0.005)	(0.006)	(0.004)	(0.006)	(0.006)
**Hofstede (*N*_dyads_ = 32,381; *N*_leaders_ = 7,604)**						
Cultural distance × Hierarchical position	0.006^†^	–0.010*	0.016**	0.007^†^	–0.010^†^	0.017**
	(0.003)	(0.005)	(0.006)	(0.004)	(0.006)	(0.006)
**Schwartz (*N*_dyads_ = 32,381; *N*_leaders_ = 7,702)**						
Cultural distance × Hierarchical position	0.006^†^	–0.007	0.013*	0.008*	–0.010^†^	0.018**
	(0.003)	(0.005)	(0.006)	(0.004)	(0.006)	(0.006)
**Berry (*N*_dyads_ = 32,082; *N*_leaders_ = 7,547)**						
Cultural distance × Hierarchical position	0.002	–0.013**	0.015**	0.006^†^	–0.012*	0.018**
	(0.003)	(0.005)	(0.006)	(0.004)	(0.005)	(0.006)
**Beugelsdijk (*N*_dyads_ = 28,428; *N*_leaders_ = 6,750)**						
Cultural distance × Hierarchical position	0.003	–0.007	0.010	0.008^†^	–0.008	0.016*
	(0.004)	(0.006)	(0.006)	(0.004)	(0.006)	(0.007)
**Hofstede and Schwartz (*N*_dyads_ = 32,232; *N*_leaders_ = 7,574)**						
Cultural distance × Hierarchical position	0.006^†^	–0.007	0.013*	0.007^†^	–0.008	0.015*
	(0.003)	(0.005)	(0.006)	(0.004)	(0.006)	(0.006)
**GLOBE, Hofstede, and Schwartz (*N*_dyads_ = 32,232; *N*_leaders_ = 7,574)**						
Cultural distance × Hierarchical position	0.005	–0.008^†^	0.013*	0.006	–0.007	0.013*
	(0.003)	(0.005)	(0.005)	(0.004)	(0.005)	(0.006)

*Standard errors clustered at leader level. Fixed effects for constant, year, industry, nationality (leader), nationality (observer), age (leader), age (observer), age (leader) × age (observer), gender (leader), gender (observer), gender (leader) × gender (observer), hierarchical position (subordinate vs. superior), and cultural distance (leader–observer) are included but not reported. Standard errors between parentheses.*

*^†^*p* < 0.10, **p* < 0.05, ***p* < 0.01, ****p* < 0.001.*
